# Effect of different colloidal gold nanomaterials on *Ganoderma lingzhi* fermentation for production of ganoderma polysaccharide and triterpenoid through macroscopic and microscopic investigation

**DOI:** 10.1186/s12934-025-02810-0

**Published:** 2025-09-30

**Authors:** Mengqiu Luo, Muling Shi, Yang Li, Yiqing Yang, Hanqi Wei, Shengwen Luo, Wenhuan Huang, Yida Deng, Gao-Qiang Liu

**Affiliations:** 1 Key Laboratory of Pico Electron Microscopy of Hainan Province, School of Materials Science and Engineering, State Key Laboratory of Tropic Ocean Engineering Materials and Materials Evaluation, Hainan University, Haikou, 570228 Hainan Province China; 2 Hunan Provincial Key Laboratory of Forestry Biotechnology, College of Life Science and Technology, Central South University of Forestry & Technology, Changsha, 410004 Hunan Province China; 3 Molecular Science and Biomedicine Laboratory, State Key Laboratory of Chemo/Biosensing and Chemometrics, College of Chemistry and Chemical Engineering, College of Biology, Hunan University, Changsha, 410082 China

**Keywords:** Gold nanomaterials, Submerged fermentation, Fungi, Polysaccharide, Triterpenoid, Scanning electronic microscopy

## Abstract

*Ganoderma lingzhi (G. lingzhi)* is a *Basidiomycete* macrofungus valued for its secondary metabolites with pharmacological activity. To enhance the biosynthesis of secondary metabolites, various exogenous additives have been introduced to fungal fermentation processes. Metal nanomaterials, while known to influence cellular metabolism in mammalian systems, exhibit unclear effects when applied to macrofungal cultivation systems. The study systematically evaluates the impact of three distinct gold nanostructures—nanoparticles (AuNPs), nanorods (AuNRs), and nanoclusters (AuNCs)—on bioactive metabolite production during *G. lingzhi* submerged fermentation, employing integrated macroscopic process analytics and microscopic characterization. The results demonstrate that their impact on mycelial growth and bioactive metabolite production varied with the type, concentration, and addition timing of gold nanomaterials. Microscopic survey on cell surface morphology and nanoparticle distribution also reveals the different patterns of nanomaterial-mycelia cell interaction. Under the optimized addition conditions, AuNPs increased total polysaccharide content by 50.37% compared to the control group, while AuNRs increased triterpenoid content by 42.78%. The work confirms the potential of colloidal gold nanomaterials to facilitate the submerged fermentation of *G. lingzhi*, which is expected to encourage the development of nanomaterial additives-based approach for efficient microbial bioactive substances production.

**Graphical abstract:**

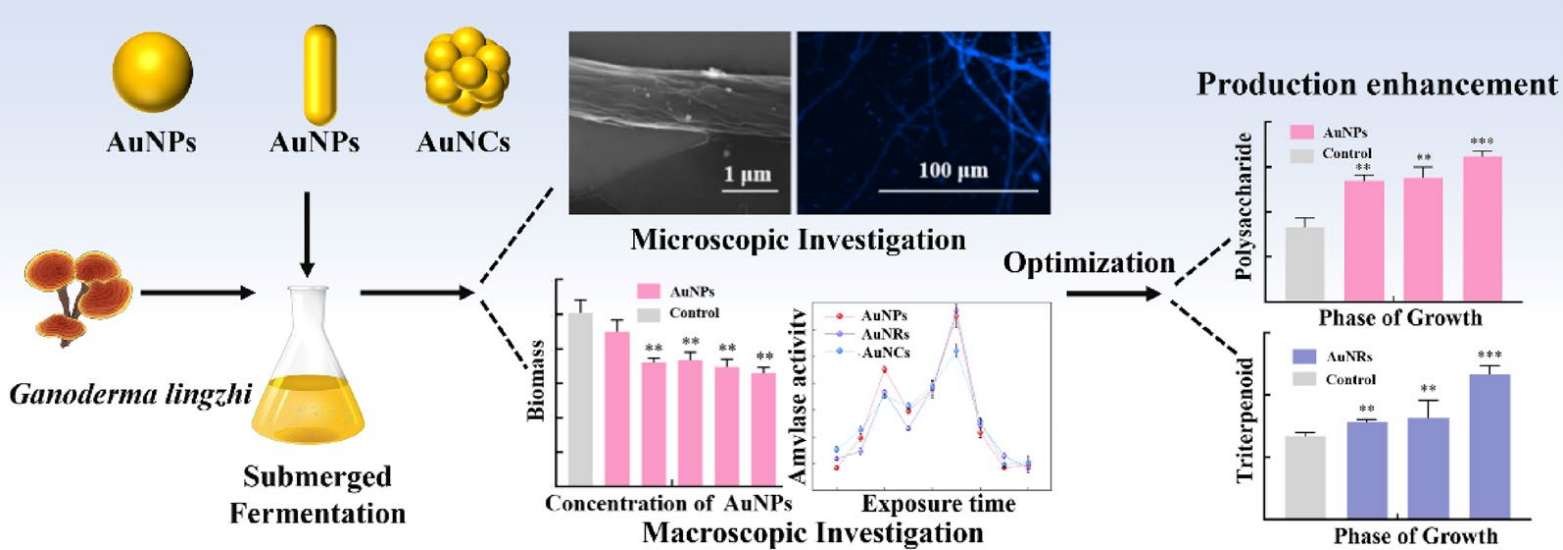

**Supplementary Information:**

The online version contains supplementary material available at 10.1186/s12934-025-02810-0.

## Introduction

*G. lingzhi* is the most widely recognized medicinal basidiomycete in Southeast Asia, with extensive research supporting its pharmacological and clinical applications. The triterpenes and polysaccharides isolated from *G. lingzhi* are considered to have numerous biological activities, including anti-cancer, anti-oxidant, anti-inflammatory response, anti-aging and regulating immunity [1]. Submerged mycelial fermentation serves as an efficient alternative to fruiting body cultivation for exopolysaccharide production, offering advantages including shorter cultivation cycles, consistent product quality, and year-round production capability [2]. Optimizing environmental conditions through manipulation of temperature [3], inoculation amount [4], pH [5], and other parameters has become a major research focus in current research [6]. Involvement of the exogenous substances in liquid submerged fermentation has also been reported to accelerate mycelial growth and metabolite production [7]. 

Nanomaterials have been introduced as exogenous additives in microbial fermentation systems in recent years, demonstrating enhanced bioproduction efficiency [8]. It has been reported that during the process of microbial fermentation for bioenergy production, nanoparticles can significantly enhance the gas–liquid mass transfer rates while also serving as carriers for exogenous enzymes [9]. Moreover, these nanocatalysts can be synthesized in an environmentally friendly manner through microbial fermentation [10]. Gupta et al. reported the *Fusarium oxysporum*-biosynthesized ZnO nanoparticles as catalysts for enhancing bioethanol production from rice straw, thereby improving ethanol yield [11]. Although these reports prove that nanomaterials can promote microbial fermentation at the macro level, the mechanism of how nanomaterials promote fermentation process at the micro level, such as cellular interaction between nanomaterials and mycelium, has not yet been described and explained in detail [12].

Current research at the intersection of nanomaterials and biological systems primarily focuses on their interactions with mammalian cells, particularly in the context of therapeutic development and toxicological assessment [13]. It has been widely recognized that mammalian cells can internalize nanoparticles through endocytosis in a morphology-dependent manner [14]. Nanomaterials can induce the generation of reactive oxygen species (ROS) in cells. Upon cellular uptake, nanomaterials interact with mitochondria and NADPH oxidase, leading to ROS production. As a specific molecular regulator of cell signaling and function, ROS brings about physiological changes that can affect cell growth and secondary metabolic biosynthesis [15].

Compared to mammalian cells with a membrane as the outer layer structure, fungal cells are covered by a cell wall which serves as a permeability barrier and protective shield, suggesting a distinctive scenario of cell-nanoparticle interaction [16, 17]. Currently, there is a considerable lack of studies on the interaction between nanomaterials and fungi as well as other species possessing cell walls. The majority of studies focusing on fungi and nanomaterials primarily concentrate on their antifungal activity and treatment of fungal infections, with few reports regarding the impact of nanomaterials on fungal liquid fermentation [18].

As stable metal nanoparticles, AuNPs exhibit high safety, biocompatibility, and facile synthesis. The unique physical and chemical properties have led to their inclusion in various biological applications, including sensors, drugs /gene delivery, photothermal therapy, and contrast agents for imaging [19]. Hence, in our work, gold nanomaterials were chosen as the subject of investigation to explore their impact on the production of secondary metabolites of G. *lingzhi* during fermentation. The typical gold nanomaterials, including gold nanoparticles (AuNPs), gold nanorods (AuNRs), and gold nanoclusters (AuNCs), exhibit distinct variations in shape and size [20–22]. It has been reported that there is a significant correlation between the size of nanoparticles and their uptake by cells [23]. The majority of nanomaterials capable of penetrating the cell membrane have diameters ranging from 10 to 100 nm [24]. Additionally, the shape also plays a crucial role in modulating cellular responses towards gold nanomaterials [25]. Therefore, in this study, three types of gold nanomaterials with distinct sizes and shapes, AuNPs, AuNRs, and AuNCs were synthesized and employed as exogenous additives for G. *lingzhi* fermentation. The impact of different addition time, types, and concentrations of gold nanomaterials on the growth of *G. lingzhi* was evaluated by the biomass yield of *G. lingzhi* mycelium and its ability to metabolize carbon sources.

Furthermore, through microscopic imaging techniques, the morphological changes on surface of the *G. lingzhi* cells during gold nanoparticle inclusion were observed. Based on these investigations, the effects of three morphologically distinct gold nanomaterials on the liquid fermentation products of *G. lingzhi* were further investigated through macroscopic indicators such as biomass and amylase activity.

This research can be a preliminary study for exploring the effect of different gold nanomaterials as novel elicitors on polysaccharide and triterpenoid production and growth of *G. lingzhi*, laying groundwork for future studies on nanomaterial-mediated fungal fermentation enhancement.

## Experimental section

### Materials

The following reagents were employed for gold nanomaterial synthesis: Tetrachloroauric (III) acid trihydrate (HAuCl_4_·4H_2_O), sodium citrate (C_6_H_5_Na_3_O_7_), sodium borohydride (NaBH_4_), sodium hydroxide (NaOH), anhydrous ethanol (C₂H₅OH), glutathione (GSH) (≥ 99%), cetyltrimethylammonium bromide​ (CTAB, C_19_H_42_BrN), silver nitrate (AgNO_3_), ascorbic acid (C₆H₈O₆). The experimental materials required for *G. lingzhi* fermentation are as follows: potato, glucose, soluble starch, corn flour, peptone, yeast extract, dipotassium hydrogen phosphate (K_2_HPO_4_), magnesium sulfate heptahydrate (MgSO_4_·7H_2_O), vitamin B_1,_ and agar powder. The chemicals utilized in this study comprised: potassium sodium tartrate, 3,5-dinitrosalicylic acid (DNS), NaOH, phenol, citric acid monohydrate (C_6_H_8_O_7_·H_2_O), trisodium citrate dihydrate (Na_3_C_6_H_5_O_7_·2H_2_O), maltose, ​​ ​​hydrochloric acid​ (HCl), sodium chloride (NaCl), potassium chloride​​ (KCl), dibasic sodium phosphate (Na_2_HPO_4_), sodium dihydrogen phosphate dodecahydrate​(NaH_2_PO_4_ ·12H_2_O); 25% glutaraldehyde; 50% ethanol; 70% ethanol; 90% ethanol; anhydrous ethanol, phosphate buffer saline (PBS). concentrated sulfuric acid (H_2_SO_4_), vanillin, acetic acid (CH_3_CO_2_H), and perchloric acid​​ (HClO_4_).

In addition, all the dispersing agents used in the experiment were ultrapure water (18 MΩ*cm). All chemicals meet ultra-high purity standards (≥ 99.99%).

### Purification of nanomaterials

The synthesis and characterization of gold nanomaterials are described in Sect. 1 of the Supporting Information. Gold nanoparticle synthesis was performed in an aseptic environment using sterile chemical reagents and a sterile ultrapure water system, with sterile products directly yielded through chemical reduction methods.

AuNPs were prepared by sodium citrate reduction method [26]. The prepared AuNPs solution was sub-packed into a 10 mL centrifuge tube and centrifuged at 6000 rpm for 15 min. After the supernatant was removed, it was redissolved with ultrapure water and repeated twice to obtain a purified AuNPs solution [27].

AuNRs were prepared by seed crystal method. The crystalline seeds of gold nanoparticles were synthesized in the first step. The AuNRs solution was centrifuged at 8000 rpm for 10 min, and the supernatant was removed and redissolved with ultrapure water. After repeating twice, the residual reagent was removed and AuNRs were successfully purified [27].

AuNCs were prepared by glutathione reduction [28]. The obtained 150 mL AuNCs solution was loaded into an 8000Da dialysis bag and purified by dialysis [29]. The purified AuNCs were collected post-dialysis.

### Strain and culture conditions

*G. lingzhi* was purchased from the China General Microbiological Culture Collection Center [30]. The fermentation medium was sterilized by autoclaving at 121 °C for 15 min before inoculation. As previously described, the mycelia of *G. lingzhi* were grown in PDA medium (200.0 g potato extract, glucose 10.0 g/L, KH_2_PO_4_ 3.0 g/L, MgSO_4_·7H_2_O 1.5 g/L, VB_1_ 0.05 g/L, agar powder 15 ~ 20.0 g/L) and placed on a constant temperature incubator at 28 °C for 7 days. The fungus seed culture medium (Glucose 35 g/L, corn flour 5 g/L, peptone 5 g/L, yeast extract 2.5 g/L, K_2_HPO_4_ 1 g/L, MgSO_4_·7H_2_O 0.5 g/L, VB_1_ 0.05 g/L) was grown in a 500 mL shake flask with keeping at 160 rpm/min at 28 °C for 7 days. The submerged fermentation medium (Soluble starch 40.47 g/L, peptone 7.86 g/L, K_2_HPO_4_ 1.5 g/L, VB_1_ 0.05 g/L) was inoculated with 10.0% (v/v) seed culture and incubated on a rotary shaker with shaking at 180 rpm/min at 28 °C for 7 days.

### Interaction of gold nanomaterials and *G. lingzhi* mycelium cells

The submerged fermentation medium was inoculated with 10.0% (v/v) seed culture, and the control group (without gold nanomaterials) and the experimental groups with different concentrations of three kinds of gold nanomaterials were set respectively. The concentration of gold nanomaterials was determined according to the initial HAuCl₄ concentration used in the synthesis process. The exposure concentrations of nanomaterials applied to *G. lingzhi* mycelial cells were selected based on previously reported nanoparticle concentrations in the literature. Each group had three parallels and was fermented at 28 °C and 180 rpm/min for 7 days.

### Determination of biomass and amylase activity

Method for determination of biomass of *G. lingzhi* mycelia: After 7 days of fermentation, the broth was separated from the culture medium using an 80-mesh sieve. The *G. lingzhi* mycelia were then rinsed three times with distilled water to eliminate residual medium components that could interfere with biomass quantification and metabolite analysis. The harvested mycelia were placed in a 60 °C drying oven and desiccated to constant weight.

The amylase activity in the fermentation medium of *G. lingzhi* was determined. The supernatants from the control group and gold nanomaterial-treated *G. lingzhi* groups were collected as crude enzyme extracts for amylase activity assays. Detailed steps are provided in the Supporting Information. (Fig. S2).

### Determination of pH and dissolved oxygen (DO)

Fermentation broth from the control group and gold nanomaterial-treated *G. lingzhi* group were collected to monitor pH and DO dynamics. The pH and DO values were determined at 0, 1, 2, 3, 4, 5, 6, 7, and 8 days of fermentation according to the instrument’s standard protocol. The pH was measured using a digital pH meter (Bel^®^, Mod. W3B), while the DO was measured with a multi-parameter portable water quality analyzer (Octadem, W-1). All samples were analyzed in triplicate to ensure measurement accuracy.

### Fluorescence microscopy imaging

Fluorescence microscope (FM), Cytation 7, USA, was applied to characterize morphological changes of *G. lingzhi* mycelia under the influence of gold nanomaterials. The fermentation broth of the control group and the three gold nanomaterials groups was centrifuged at 7000 rpm for 10 min. After the medium was removed, it was washed three times with PBS to eliminate the effect of the medium on fluorescence imaging. 10 μL PBS with dispersed mycelia was dropped onto a glass slide and covered with a cover glass to avoid bubbles. The samples were placed under a fluorescence microscope, and the mycelium were imaged using bright-field microscopy and fluorescence detection via the DAPI (4′,6-diamidino-2-phenylindole) channel, configured for 405 nm excitation.

### Electron microscopy imaging

Scanning Electron Microscope (SEM) images and elemental analysis images (EDS) were taken on the Tescan Mira4 field emission SEM. 1 mL fermentation broth of the control and the gold nanomaterial groups were washed three times with PBS. Then the samples were fixed with 2.5% glutaraldehyde at room temperature for 15 min and washed once with PBS. Subsequently, the samples were washed with 50, 70, 90, and 100% ethanol solutions for 5 min.

### The extraction and determination of polysaccharides

Centrifuge to collect the mycelium of *G. lingzhi* and rinse it with about 250 mL of distilled water. The mycelium was vacuum freeze dried until no weight change occurred. After grinding the dried *G. lingzhi* mycelium into powder, 1 g powder was soaked in 50 mL ultrapure water for 24 h. Following soaking, the extract was subjected to hot water extraction at 90 °C for 1 h, and subsequently to ultrasound-assisted extraction using a water bath sonicator (40 kHz, 100 W) at room temperature for 1 h. After treatment, the supernatant was collected by centrifugation to obtain the crude polysaccharide solution. Subsequently, 10 µL of the sample was pipetted into a dry test tube, and the volume was adjusted to 2 mL with ultrapure water. Sequentially 1 mL of 6% (w/v) phenol solution and 5 mL of concentrated sulfuric acid (98%) was added to the sample. After standing for 10 min, thorough vortex mixing was conducted, and the mixture was allowed to cool to room temperature (25 °C). Absorbance (A) at 490 nm was measured using a spectrophotometer. The content of *G. lingzhi* crude polysaccharides was calculated by glucose standardization. See the detailed standard curve determination method in the supporting information (Fig. S2).

### The extraction and determination of triterpenoids

1 g of *G. lingzhi* mycelia powder was soaked in 50 ml of absolute ethanol for 24 h. ​At the end of soaking, the samples were extracted in a water bath at 75 ℃ for 1 h, followed by ultrasonic shaking for 1 h. After treatment, the mixture was centrifuged at 8000 rpm for 10 min, and the supernatant was collected and mixed well as the sample for triterpenoids determination. 0.4 mL of a 5% vanillin/glacial acetic acid (w/v) solution and 1.0 mL of a perchloric acid solution were added to 500 µl extracts then incubated at 60 ℃ for 15 min, and cooled to room temperature. The absorbance of the sample was quantified at 548 nm using a spectrophotometer after 10 mL of glacial acetic acid was added. The triterpenoid content in *G. lingzhi* was quantified using an ursolic acid standard curve (R² >0.99), with detailed calibration methodology provided in the supporting information (Figure S3).

## Results and discussion

### Characterization of gold nanomaterials

**Fig. 1:**
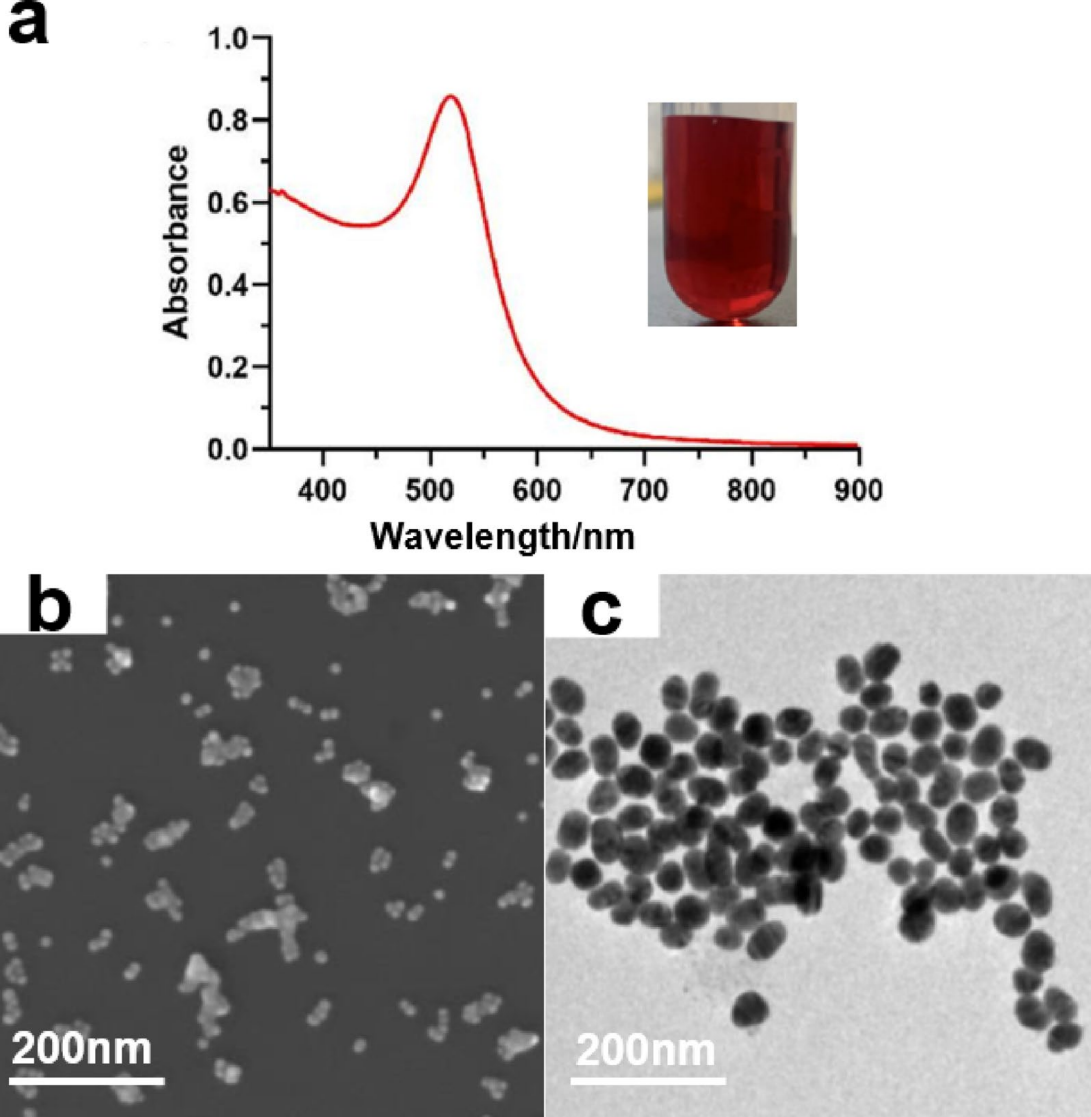
**a** UV-vis absorption spectrum of AuNPs. The color of gold nanoparticles in sunlight. **b** SEM of AuNPs. **c** TEM of AuNPs

In this study, AuNPs were synthesized using an adapted citrate reduction method [26]. The UV-vis spectra for the AuNPs in Fig. 1a demonstrate an absorption peak at approximately 520 nm for the AuNPs [31]. The SEM and TEM images in Fig. 1b and c exhibit high monodispersity and purity in terms of morphology. The measured size is approximately 30 nm, which is consistent with the morphological dimensions reported in the literature [32].

**Fig. 2:**
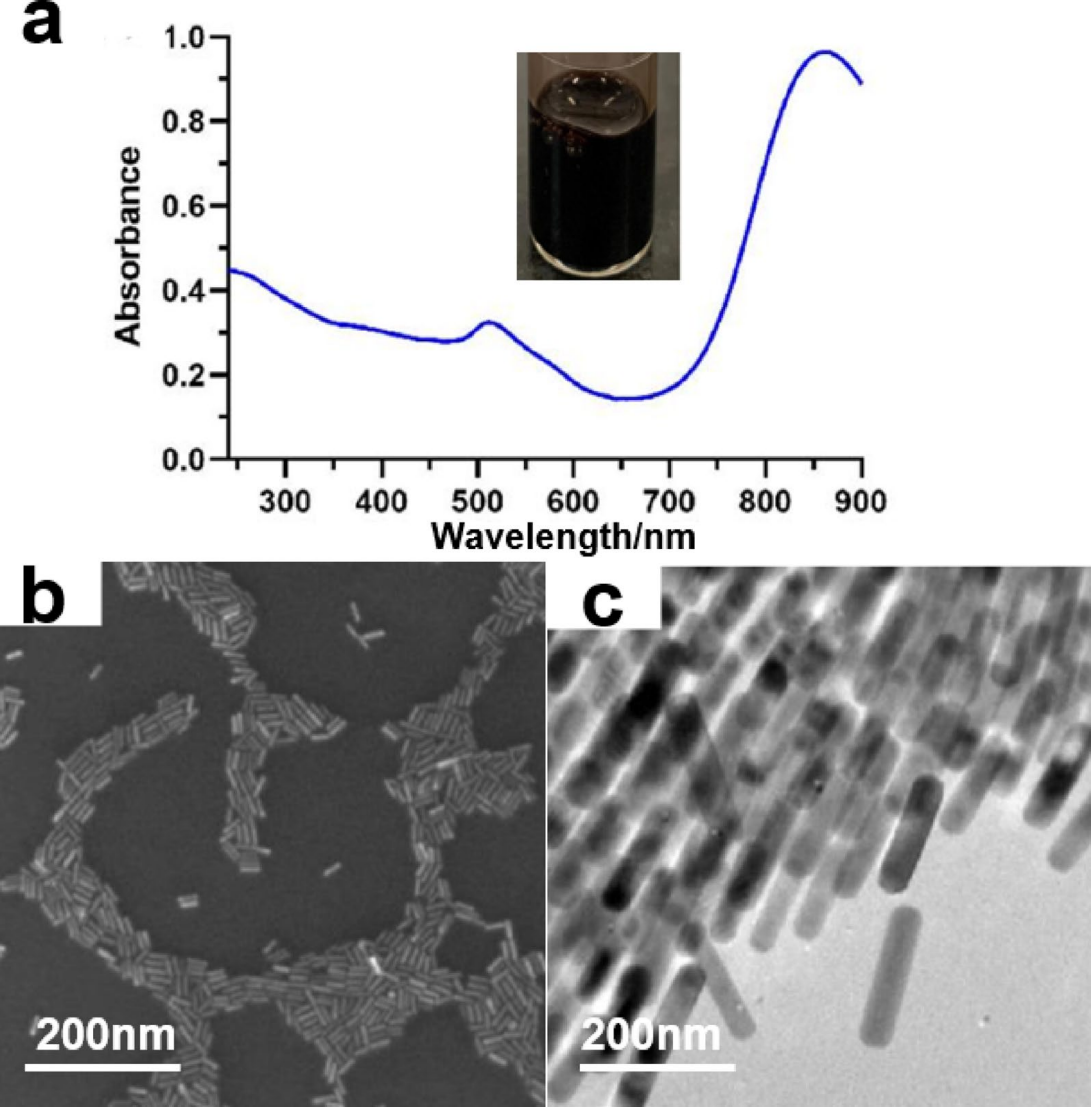
**a** UV-vis absorption spectrum of gold nanorods. The color of AuNRs in sunlight. **b** SEM of AuNRs. **c** TEM of AuNRs

The AuNRs were synthesized using a seed-mediated method in combination with the protocols developed by Jana et al. and Nikoobakht et. al [33, 34]. The UV-vis spectra of AuNRs in Fig. 2a exhibit a lateral absorption peak at 512 nm, which is observed in addition to a longitudinal absorption peak at 820 nm. As shown in Fig. 2b, c the SEM and TEM characterization results of AuNRs show that the particle shape closely resembles a cylinder, with a rounded end, consistent with findings reported in the literature [35]. The AuNRs exhibit high monodispersity, displaying a homogeneous morphology with a length of approximately 100 nm and a width of around 30 nm. Notably, the TEM characterization shows purely nanorod-shaped morphology (Fig. 2c) with absence of spherical impurities, confirming the high purity of the as-prepared gold nanorods, which aligns with the results of seed-mediated synthesis documented in previous studies [34].

**Fig. 3:**
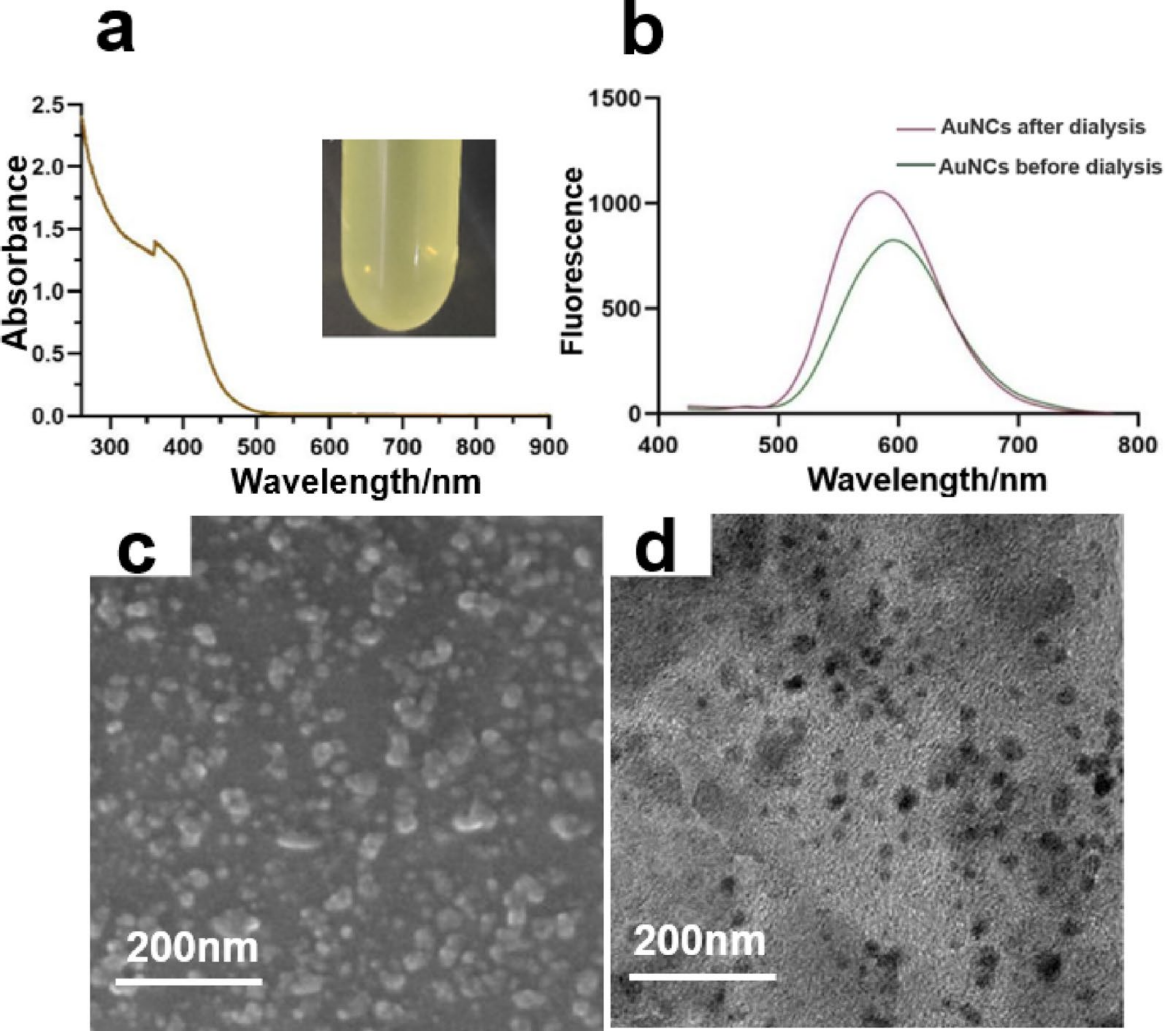
**a** UV-vis absorption spectrum of AuNCs. The color of AuNCs in sunlight. **b** Fluorescence spectra of AuNCs before and after dialysis. **c** SEM of AuNCs. **d** TEM of AuNCs

The AuNCs were synthesized using the template method as described by Luo Zhen-Tao et al. [28]. The UV − vis spectra depicted in Fig. 3 showed an absorption peak at approximately 360 nm for the AuNCs. The TEM images demonstrate the AuNCs showed good dispersibility in water, with a size range of about 2 to 5 nm. (Fig. 3d) The AuNCs exhibited bright orange fluorescence under the irradiation of 365 nm ultraviolet light. The fluorescence spectra of AuNCs were obtained at 404 nm excitation, and the result exhibited a prominent peak at around 600 nm (Fig. S4, 5).

Nanomaterial preparation might produce an inhomogeneous size distribution and involve excess reductant, which might impede the growth of *G. lingzhi* [36]. Therefore, it is necessary to apply a purification process after nanomaterials preparation. The purification methods employed in this paper vary according to the distinct preparation approaches and properties of the three nanoparticles. The feasibility of centrifugation for the separation of colloidal particle mixtures was demonstrated by Sharma et al. [2, 7]. who successfully separated nanoparticles of various shapes and sizes through this method. Therefore, The AuNPs and AuNRs were purified through centrifugation to eliminate excess reductant. The UV-vis absorption spectra of AuNRs were measured both before and after purification. and a blue shift of approximately 4 nm was observed. which infers the effective separation of distinct nanomaterial morphologies (Fig.S3) [36]. The AuNCs were purified through dialysis. The fluorescence spectra revealed that the purified clusters exhibit an enhanced fluorescence signal (Fig. 3b), which aligns with the findings reported by the Guan group [29]. The present study involved the synthesis and purification of three distinct gold nanomaterials, which were subsequently characterized using various analytical techniques including UV-vis, SEM, TEM, and FM, among others. The results demonstrate that the synthesized materials exhibit uniform dispersion, homogeneous morphology, and no significant aggregation.

The AuNPs used in the study have a diameter of 30 nm, while the AuNRs have a length of 30 nm, they share similar sizes and distinct morphology. On the other hand, both the synthesized AuNPs and AuNCs exhibit a spherical morphology, but they have different sizes. Based on this, we subsequently conducted a comparative analysis of materials with similar dimensions but varying shapes, as well as materials with similar shapes but different sizes, to investigate the impact of gold nanomaterials’ size and morphology on the growth and fermentation products of *G. lingzhi.*

### Effect of gold nanomaterials addition on the biomass of *G. lingzhi*

The impact of nanomaterials on microbial fermentation biomass has been reported in yeast cells [1, 37]. Biomass serves as an important indicator of *G. lingzhi* growth status. The dynamic changes in *G. lingzhi* biomass, such as the increment in mycelial dry weight, can be used to evaluate its vitality or the efficiency of the cultivation system. The seed culture of *G. lingzhi* was transferred into bottles, and three types of gold nanomaterials with varying concentrations were added as the experimental group, the control group was established without the addition of gold nanomaterials. After 7 days of fermentation and cultivation, the mycelium was separated from the culture medium, and the weight of the dried mycelium represented the biomass [38].

**Fig. 4:**
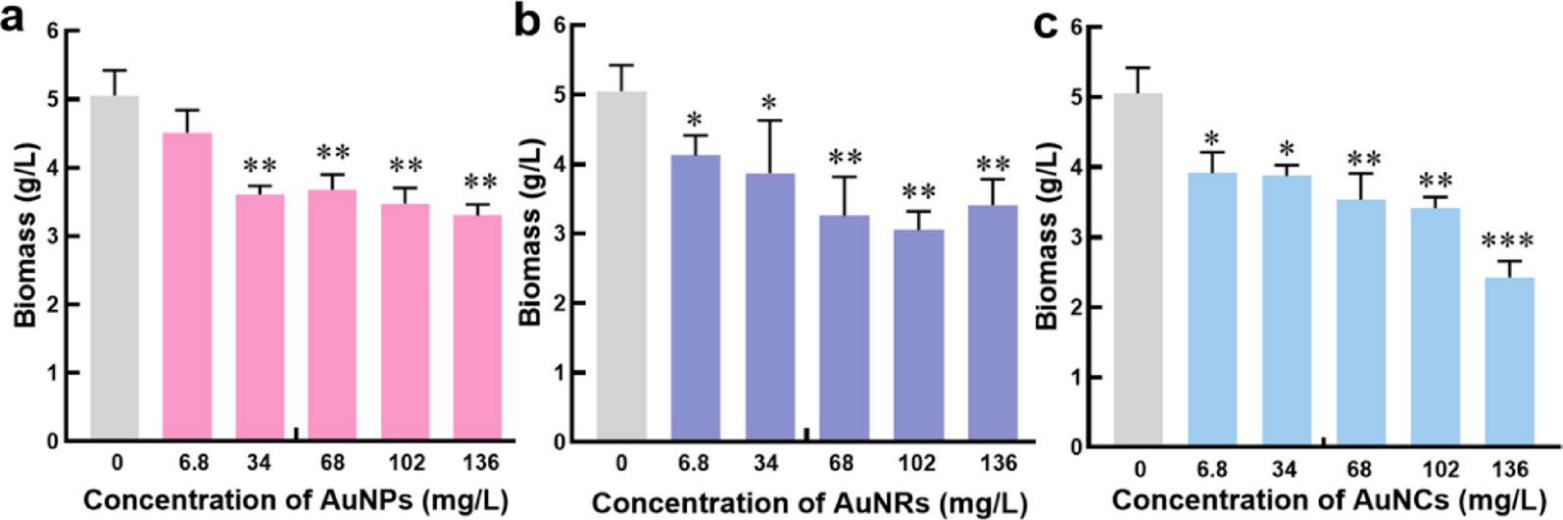
**a** The relationship between AuNPs concentration and biomass. **b** The relationship between AuNRs concentration and biomass. **c** The relationship between AuNCs concentration and biomass. The data in the figure is the mean ± standard error, * Stands for *P*<0.05, ** stands for *P*<0.01,*** stands for *P*<0.001

Figure 4 shows the changes in biomass with varying concentrations of three types of gold nanomaterials. The control group exhibited a biomass of 5.05 g/L for *G. lingzhi*. At an AuNPs concentration of 6.8 mg/L, there was no significant disparity in biomass between the experimental and control groups. However, when concentration of AuNPs is higher than 6.8 mg/L, the biomass decreases and remains at approximately 3.51 g/L, which indicates significant mycelium growth inhibition.

The same methodology was employed to study the impact of AuNRs on biomass. AuNRs exhibited inhibitory effects on the growth of *G. lingzhi* mycelium, with the lowest biomass observed in the 102 mg/L AuNRs group at only 3.05 g/L. The impact of AuNCs on biomass was further investigated. The addition of AuNCs also inhibited the growth of *G. lingzhi* mycelium, and the inhibitory effect became more pronounced with increasing concentration. In the group treated with 136 mg/L AuNCs, the biomass of *G. lingzhi* mycelium was only 2.42 g/L at minimum.

In summary, the incorporation of three types of gold nanomaterials led to a reduction in the biomass produced during liquid fermentation of *G. lingzhi*. Although both the AuNPs and AuNRs groups exhibited inhibitory effects on the growth of *G. lingzhi* mycelium, *G. lingzhi* mycelium biomass in each group remained above 3 g/L. The lowest *G. lingzhi* mycelium biomass was recorded in the 136 mg/L AuNCs group, with only 2.42 g/L. The inhibitory effect was pronounced, potentially attributed to the perturbation of biosynthetic processes associated with cell growth due to elevated levels of ROS following nanomaterial addition [39]. Moreover, gold nanomaterials are reported to possess antifungal activity by directly interacting with proteins and enzymes followed by conformational changes, that lead to the loss of their normal function and further impede fungal cell growth Consequently, the biomass of the gold nanomaterial group was lower compared to the control group [40, 41].

### Effect of gold nanomaterials addition on carbon source utilization, pH and DO during *G. lingzhi* fermentation process

Carbon source utilization is a pivotal metabolic process in *G. lingzhi.* Starch in the culture medium serves as a crucial carbon source for *G. lingzhi* mycelial growth. During fermentation, *G. lingzhi* requires amylase to hydrolyze starch and produce soluble sugar molecules for nutritional assimilation. The depletion of starch in the medium or the accumulation of toxic by-products leads to decline in enzyme activity [2].Therefore, we measured amylase activity to evaluate carbon source utilization, thereby investigating the effects of gold nanoparticles on fungal metabolism. The seed culture of *G. lingzhi* was transferred into bottles, and both a control group and an experimental group with three types of gold nanomaterials were established. The fermentation broth of *G. lingzhi* should be sampled daily for a period of 8 consecutive days, and after centrifugation, the supernatant was obtained as a crude enzyme solution. Following treatment, the maltose content was determined using the maltose standard curve formula, while amylase activity was calculated using the formula in supporting information.

**Fig. 5:**
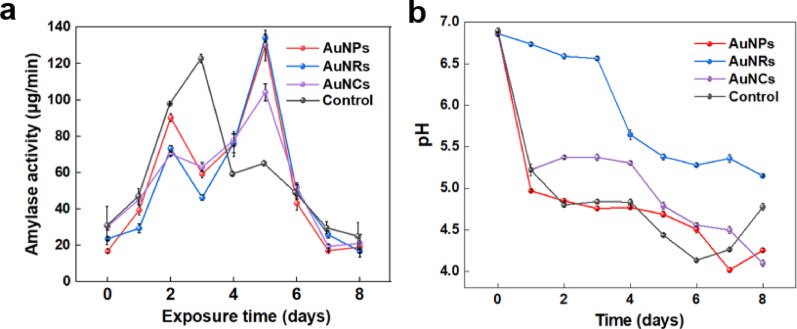
**a** Changes of amylase activity in *G. lingzhi* fermentation broth. The addition concentrations for all three types of gold nanomaterials were all set at 136 mg/L. All data points represent the mean ± SD of three independent technical replicates. **b** Changes of the pH in *G. lingzhi* fermentation broth. The addition concentrations for all three types of gold nanomaterials were all set at 136 mg/L. All data points represent the mean ± SD of three independent technical replicates

The changes in enzyme activity in the control group and the groups supplemented with AuNPs, AuNRs, and AuNCs for 8 days are respectively depicted in Fig. 5a. In the control group, enzyme activity showed a tendency to increase and then decrease, reaching its peak on Day 3. Similarly, the three nanomaterials exhibit comparable trends with enzyme activity peaking on Day 5.

The addition of gold nanomaterials delayed the time of amylase reaching the peak in the fermentation broth of *G. lingzhi*. With the increase of culture time, the enzyme activity gradually decreased [42]. Time delay in enzyme activity reaching its peak after the addition of gold nanomaterials indicates a delayed substrate consumption ability in mycelium, which could be one of the contributing factors to the reduction in mycelium biomass observed in the gold nanomaterial group.

The pH value is a crucial indicator in the fermentation process, and its decrease is primarily attributed to the production of organic acids during fermentation [5]. As shown in Fig. 5b, the effect of the addition of gold nanomaterials on fermentation process was explored by comparing pH changes between AuNPs/AuNRs/AuNCs-addition groups and the control group without gold nanomaterials. The pH changes in all groups showed similar trends, exhibiting a rapid decline in pH during the initial stage, followed by comparative stability throughout the middle and late stages. Similar pH kinetics have been reported by Liu et al. [43]. It is worth noting that the addition of AuNRs resulted in a delay in the onset of rapid pH decline, a lower pH change rate, and a higher minimum pH during fermentation. This may be attributed to cellular damage caused by AuNRs, which affects the production of organic acids [44].

We then monitored the dissolved oxygen (DO) concentration changes over fermentation time. There was a consistent DO trend in the three types of gold nanomaterial addition groups and the control group without gold nanomaterial addition, as shown in Figure S4. But the DO consumption was slightly slower in gold nanomaterial addition groups than control group. The reason may be that the surface charge and chemical properties of the gold nanomaterials inhibit the respiratory chain, thereby resulting in a slower DO consumption rate [45].

In summary, three types of gold nanomaterials were cultured alongside *G. lingzhi* strains to measure their biomass and carbon consumption. The findings indicate that gold nanomaterials impede mycelium growth, influence substrate carbon source utilization and influence pH value. This might be because when nanomaterials enter cells or organisms, complex interactions happen between their surface and the internal components of the organism, like proteins, carbohydrates, fatty acids, etc. Despite these interactions, the organism maintains favorable growth conditions overall. Therefore, in subsequent experiments, we employed microscopic imaging to characterize the interaction between nanomaterials and mycelial cells.

### Fluorescence microscopic observation of the interaction between gold nanomaterials and *G. lingzhi* mycelia cells

Microscopic imaging has the advantages of high resolution, deep-tissue imaging and fast measurement, which can more intuitively characterize fungal mycelia and cells [46]. Therefore, it could be instructive to further investigate the impact of gold nanomaterials on the microscopic morphological alterations of *G. lingzhi* hyphae.

Due to the pronounced light scattering characteristics exhibited by nanomaterials, achieving imaging through ordinary optical microscopes under white light illumination becomes challenging. It is reported that the presence of chitin, sulfur-binding proteins, and melanin in the cell wall of fungi emit autofluorescence light when excited by UV and blue light [47]. Moreover, it is worth noting that cysteine itself exhibits autofluorescence. Hence, we opted for fluorescence microscopy to visualize *G. lingzhi* incubated with gold nanomaterials, taking the advantage of autofluorescence of mycelium under UV light irradiation [48].

The seed culture of *G. lingzhi* was transferred into bottles, and both the blank control group and the experimental group with three types of gold nanomaterials were established. After harvesting the mycelium fermentation broth and removing the culture medium, the mycelium was examined using a fluorescence microscope in both bright field (BF) and DAPI channels.

**Fig. 6:**
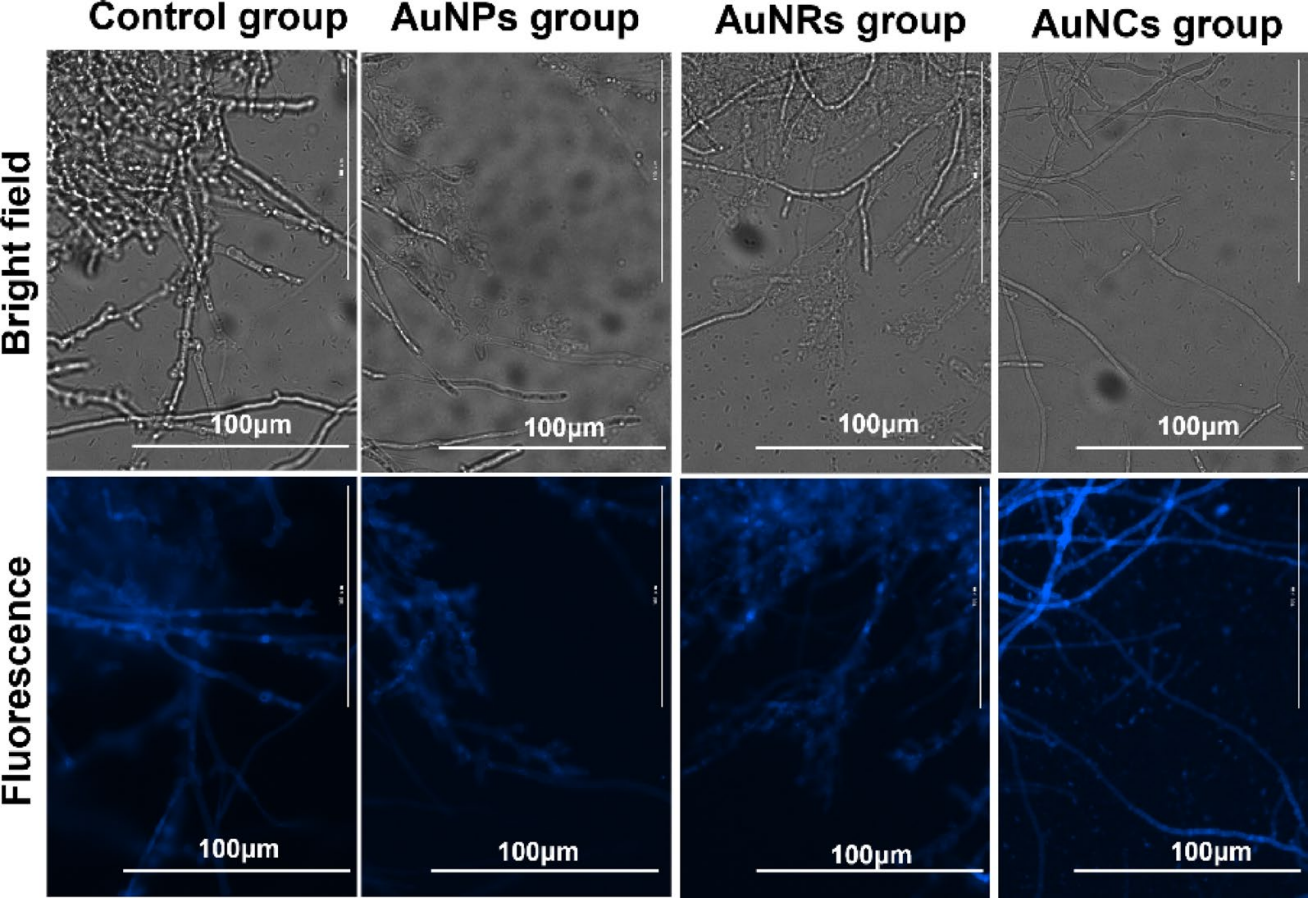
The bright field diagram and fluorescence imaging diagram of the control group and the gold nanomaterial groups (The scale bar is 100 μm)

The fluorescence imaging (FI) results depicted in Fig. 6 illustrate that both the mycelium in the AuNPs group and the control group showcase filamentous morphology with sleek surfaces. Notably, mycelium cultivated in an incubator shaker exhibits a spherical morphology characterized by densely dispersed mycelium and the majority of the mycelium is intricately intertwined, while isolated mycelia are rarely detected.

The mycelium in the AuNPs group displayed unaltered morphology under both BF and FI, suggesting that AuNPs did not induce significant damage to the mycelium. This finding can be ascribed to the inherent low toxicity of AuNPs [14], which minimally affected mycelium viability. The mycelium in the AuNRs group was more intertwined, making it difficult to image individual mycelium. However, no significant alterations in the morphology of the mycelium within the AuNRs group were observed compared to the control group. This suggests that while AuNRs effectively inhibited mycelium growth, they did not induce mycelial rupture. The FI of AuNCs group exhibited higher activity and clearer fluorescence imaging in mycelium compared to the control group and the first two gold nanomaterial groups. However, due to the presence of fluorescence from AuNCs, the background view was more complex than that of other groups. Fluorescence imaging results show that the gold nanomaterials exhibited no detrimental effects on the mycelium of *G. lingzhi*.

### Scanning electronic microscopic survey on cell surface morphology and nanoparticle distribution

The magnification of SEM surpasses that of an optical microscope by more than 30,000 times, enabling clearer characterization of the microstructure of substances. To further investigate the impact of gold nanomaterials on mycelium surface morphology, we employed an SEM for observation.

After removing the supernatant by centrifugation, the mycelium fermentation broth from both the control and experimental groups containing three types of nanomaterials was fixed with glutaraldehyde. Subsequently, PBS was used for centrifugal cleaning followed by gradient elution. The eluted mycelium and anhydrous ethanol mixture were then dripped onto a silicon chip which was fixed on the sample platform using conductive glue. Finally, platinum spraying was carried out to observe the status of gold nanomaterials and mycelium under SEM.

**Fig. 7:**
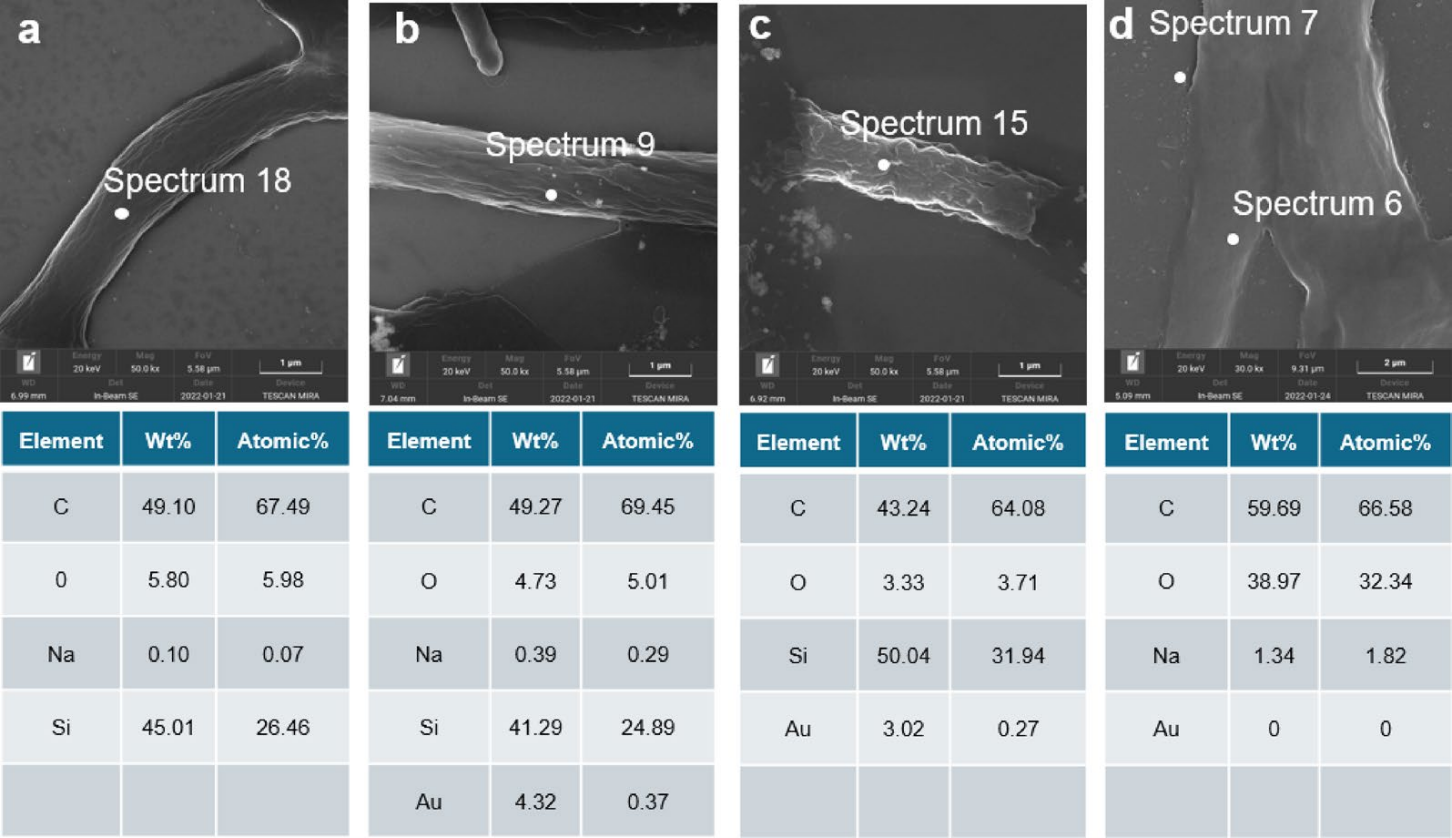
Scanning Electron Microscopy (SEM) and elemental analysis (EDX) of the interaction between gold nanomaterials and *G. lingzhi* mycelium **a** SEM image of *G. lingzhi* mycelium. Elemental composition of Spectrum 18 site was shown in lower panel. **b–d** SEM image and elemental analysis of *G. lingzhi* mycelium and AuNPs/AuNRs/AuNCs interaction, respectively. Where Wt% denotes Weight% and Atomic% represents Atomic Percent

Figure 7a illustrates that the surface of the *G. lingzhi* mycelium of the control group appears smooth without any visible attachments, indicating healthy and plump growth. At lower magnification (Figure S7), although some winding is observed in the mycelium, the overall state was still good, the mycelial network appears ​​densely interwoven, exhibiting ​​uniform hyphal diameters​​ and plump morphology. The SEM image of the AuNPs group reveals tiny particles attachment on mycelium, which are speculated to be AuNPs. However, no significant differences in mycelium condition are observed compared to the control group. Further magnification observation confirms particle substances attached to the mycelium surface (Fig. 7b).

The elemental analysis of sites on the mycelium cell surface reveals that in the control group, spectrum18 primarily consists of C and O elements with a small amount of Na element present; however, Si element predominantly originates from silicon substrate rather than being inherent to the mycelium. *G. lingzhi* mycelium mainly comprises C and O elements (Fig. 7a). The signals in EDS originate from atoms within the surface layer of the sample; therefore, only the elemental distribution on the sample surface can be detected. Elemental analysis of the AuNPs group revealed the presence of metallic Au alongside C and O elements originating from the mycelium components, confirming the attachment of AuNPs to the surface of *G. lingzhi* mycelium.

The SEM image of the AuNRs group revealed the presence of particles adhered to the mycelium surface. In comparison with both the control group and the AuNPs group, *G. lingzhi* mycelium in the AuNRs group exhibited a bumpy surface characteristic. Upon observing a wider field of view under electron microscopy, it was observed that most of the mycelium appeared as flat and covered with numerous shiny aggregates. These mycelia are so intertwined that they are hard to separate. Elemental analysis mapping and content determination results confirmed detection of C, O, and Au elements within this region, further validating that these particles were indeed AuNRs and confirming their interaction with mycelium at this specific location (Fig. 7c).

The SEM image of the AuNCs group revealed that, unlike the other two gold nanomaterials, no particle or aggregate adsorption was observed on the mycelium surface. This observation may be attributed to the AuNCs with a small size of 2 ~ 5 nm, which falls below the resolution limit of our limited magnification. At low magnification, no aggregation of AuNCs was observed in the AuNCs group, indicating that the surface of the mycelium presents itself as smooth. In comparison to the AuNRs group, the mycelium morphology appeared fuller, while no significant difference was noted in the control group, suggesting that AuNCs induced less damage to the mycelium surface. Interaction sites between AuNCs of mycelium cannot be visually judged. After multiple determinations, we ultimately selected Spectrum 6 site in Fig. 7c and the Specturm 7 site located adjacent to the mycelium but not on its surface for elemental analysis. The detection results indicated that elements contained in Spectrum6 site were consistent with previous measurements from control group mycelia without any detectable presence of gold (Au) element; However, analysis of Specturm 7 identified a small amount of gold (Au) element confirming presence of AuNCs within culture medium (Table S7). Nevertheless, due to the size of AuNCs being only 2–5 nm which is smaller than cell wall gaps of approximately 5.8 nm, it may lead to their entering the *G. lingzhi* cells for AuNCs particles, thereby making them undetectable on the mycelium surface [49].

The SEM results revealed the adsorption of AuNPs and AuNRs on the mycelium surface. In comparison to the control group, the morphology of mycelium in the AuNPs group remained unchanged, possibly due to their lower toxicity, resulting in minimal damage to mycelium [14]. However, the mycelium surface in the AuNRs group exhibited poor condition, which could be attributed to toxic effects caused by CTAB residues present on the surface of AuNRs [37, 50]. Despite removing a portion of CTAB through centrifugation before experimentation, residual CTAB still inflicted certain levels of damage upon mycelium. Conversely, under SEM observation, mycelium morphology appeared normal and healthy in the presence of AuNCs similar to that observed in control group samples. Nevertheless, due to their small size, interaction sites between AuNCs and mycelium could not be determined through SEM or elemental analysis.

Based on the findings of Louise et al., it is postulated that AuNCs may potentially penetrate mycelium [49]. According to the Hard and Soft Acids and Bases (HSAB) theory, gold’s classification as a soft acid, it could strongly interact with sulfur-containing proteins present in the soft base membrane or phosphorus-containing bases within DNA, thereby impeding their normal synthesis, repair, and replication functions and ultimately leading to a reduction in *G. lingzhi* mycelium biomass [37]. Consequently, it is hypothesized that AuNCs exhibit a more pronounced inhibitory effect on biomass compared to AuNPs and AuNRs [10, 40].

### Effect of the type of gold nanomaterials on the yield of polysaccharides and triterpenoids

Nanoparticles used as biocatalysts can enhance heat and mass transfer rates, enzyme activity, and bioenergy conversion in microbial liquid fermentation. In addition, the addition of nanomaterials has been proven to effectively increase ethanol production in mycelia fermentation [9]. The significant impact of nanomaterials with the same elemental composition but varying morphologies and sizes on mycelia growth and mycotoxin biosynthesis has been experimentally demonstrated [1]. To investigate the influence of different morphologies and sizes of gold nanomaterials on fermentation products of *G. lingzhi*, three types of gold nanomaterials were introduced after 7 days of *G. lingzhi* fermentation. Subsequently, the samples were dried, ground into powder using a mortar, collected in 2 mL centrifuge tubes, and analyzed for the contents of polysaccharides and triterpenoids.

**Fig. 8:**
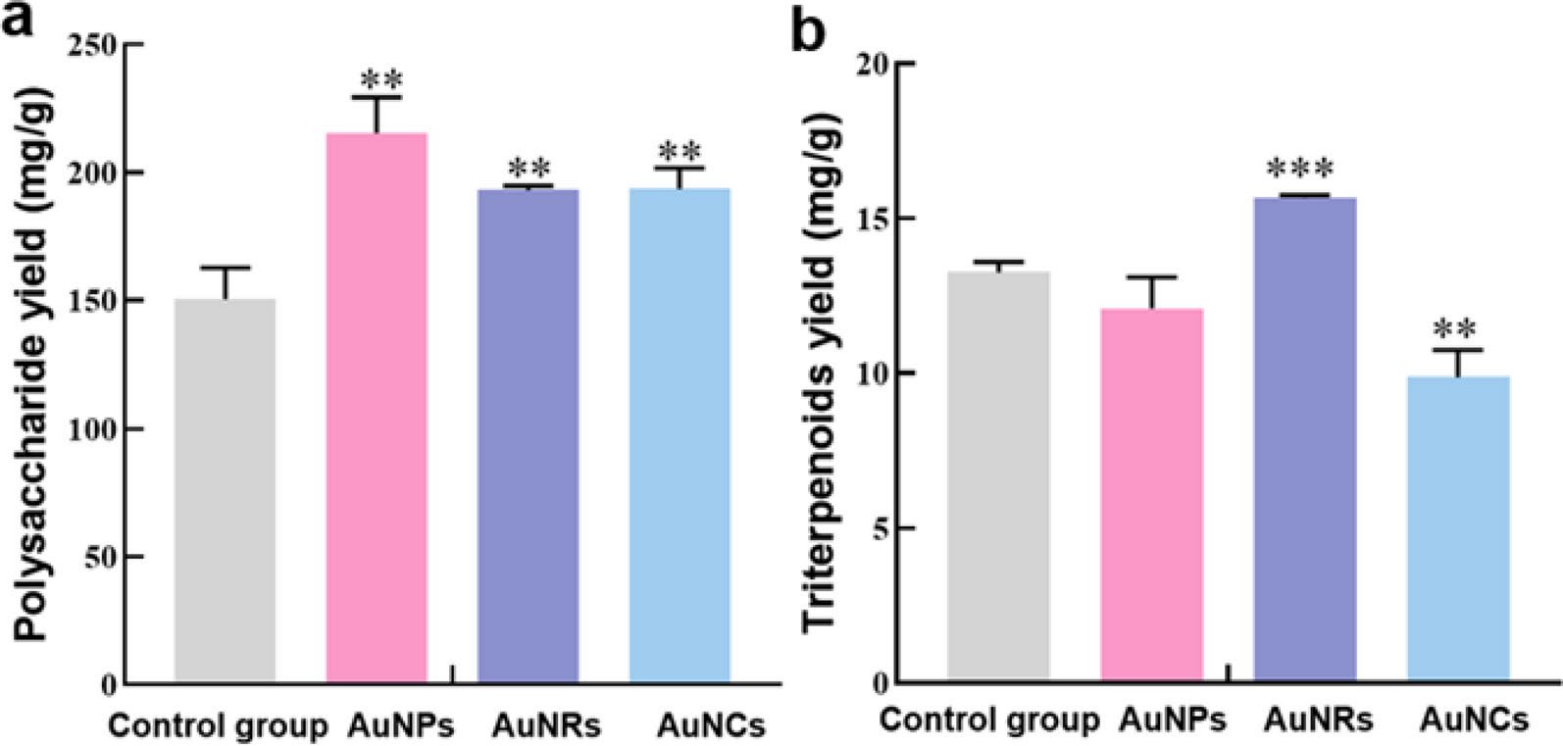
Effects of different kinds of gold nanomaterials on* G. lingzhi *fermentation products. **a** Polysaccharide yield. **b** Triterpene yield. The data in the figure is the mean ± standard error, * Stands for *P*<0.05, ** stands for *P*<0.01,*** stands for *P*<0.001

By comparing the data presented in Fig. 8, it was observed that all three gold nanomaterials significantly enhanced the yield of *G. lingzhi* polysaccharide. In comparison to the control group, the AuNPs group exhibited a remarkable increase in polysaccharide yield by 30.16%, while the AuNRs and AuNCs groups demonstrated increases of 22.10% and 22.29%, respectively. Compared to *G. lingzhi* polysaccharide, the promotional effect of gold nanomaterials on G. *lingzhi* triterpenoid content was significantly diminished. Among the three gold nanomaterials, only the AuNRs group exhibited a promotive effect on *G. lingzhi* triterpene content, while the others demonstrated an inhibitory effect (Fig. 8b).

After a comprehensive analysis, all three gold nanomaterials were found to have an impact on the content of *G. lingzhi* polysaccharides and triterpenoids, albeit with different trends of influence. In the case of AuNPs and AuNRs, which have similar sizes but different shapes, the AuNPs group exhibited a more significant promotion effect on polysaccharide yield, while the AuNRs group showed an inhibitory effect.

However, concerning *G. lingzhi* triterpenoid content, the AuNPs group displayed an inhibitory effect whereas the AuNRs group demonstrated a promoting effect. On the other hand, in spherical AuNPs and AuNCs, the promotion effect of the AuNPs group on polysaccharide yield was significantly greater than that of the AuNCs group; both groups tended to inhibit *G. lingzhi* triterpenoid content. Notably, this inhibition effect was more pronounced in the AuNCs group.

The results demonstrated that the incorporation of distinct gold nanomaterials led to varying yields of polysaccharides and triterpenes in *G. lingzhi*. This phenomenon can be attributed to the disparity in the morphological characteristics of gold nanomaterials. For instance, spherical nanoparticles exhibit the shortest phagocytic time compared to other nanomaterials, while rod-shaped particles with nearly identical volumes demonstrate enhanced cellular uptake in comparison to cylindrical particles.

### Effect gold nanomaterials concentration on the yield of polysaccharides and triterpenoids

The impact of nanomaterial addition concentration on mycelium growth has been reported. For instance, iron nanoparticles exhibit both positive and negative effects on mycelium growth and biosurfactant production. At a concentration of 1 mg/L, mycelium experiences a 57% increase in growth, while biosurfactant production increases by 63%. However, when the concentration of iron nanoparticles is increased to 1 g/L, the mycelium growth rate decreases by 77%, with no detectable biosurfactant [51].Therefore, in this study, we selected three gold nanomaterials with different Au atom addition concentrations of 6.8 mg/L, 34 mg/L, 68 mg/L, 102 mg/L, and 136 mg/L for cultivation in fermentation broth of *G. lingzhi*. Each experiment included three parallel samples to determine the content of polysaccharides and triterpenoids through sample drying.

**Fig. 9:**
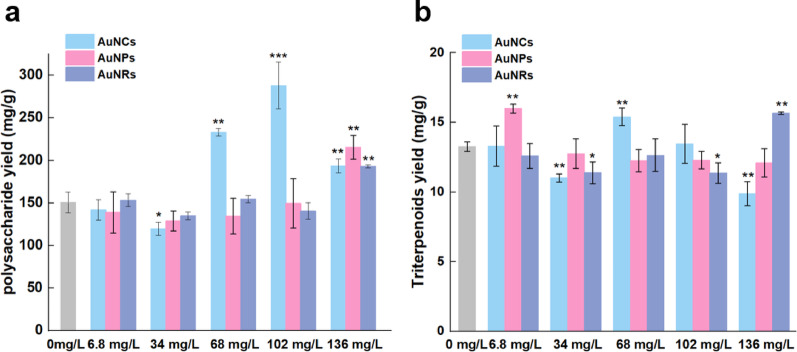
Effect of gold nanomaterial concentration on the fermentation products of *G. lingzhi.* **a** polysaccharide yield. **b** Triterpenoids yield. The data in the figure is the mean ± standard error, * Stands for *P*<0.05, ** stands for *P*<0.01, *** stands for *P*<0.001

In Fig. 9a, the control group exhibited the *G. lingzhi* polysaccharide contents at 150.42 mg/g, and no significant difference was observed between the control group and the AuNPs group at low concentrations. However, upon reaching a concentration of 136 mg/L, the AuNPs group demonstrated an increased content of *G. lingzhi* polysaccharides to 215.37 mg/g, representing a remarkable enhancement of 30.16% compared to that in the control group.

The presence of AuNRs exhibited a significant impact on the fermentation products only at elevated concentrations. Specifically, when the concentration of AuNRs was 136 mg/L, it resulted in the highest yield of *G. lingzhi* polysaccharides at 193.07 mg/g, representing a remarkable increase of 22.09% compared to the control group (150.42 mg/g).

When the concentration of AuNCs was below 68 mg/L, the presence of AuNCs inhibited the production of *G. lingzhi* polysaccharides, with a minimum yield of 119.72 mg/g observed at a concentration of 34 mg/L. Conversely, when the concentration exceeded 68 mg/L, there was an overall enhancement in *G. lingzhi* polysaccharide yield. The maximum yield of 287.97 mg/g was achieved at a concentration of 102 mg/L, representing a significant increase of 47.77% compared to the control group (150.42 mg/g).

As shown in Fig. 9b, the triterpenoids content in the control group was 13.25 mg/g, whereas, with an AuNPs concentration of 6.8 mg/L, the triterpene content in *G. lingzhi* increased to 15.98 mg/g, exhibiting a significant increase of 17.08% compared to the control group. However, at the AuNPs concentration higher than 6.8 mg/L, there was no significant difference in triterpenoids content between the treatment and control groups, both maintaining levels around 12 mg/g.

When AuNRs were added at a concentration of 136 mg/L, the triterpene content in *G. lingzhi* reached its highest value of 15.66 mg/g, exhibiting a significant increase of 15.39% compared to the control group (13.25 mg/g).

When the concentration of AuNCs was 68 mg/L, the yield of triterpenes reached its peak value at 15.38 mg/g, exhibiting a significant increase of 13.85% compared to the control group (13.25 mg/g). The addition of AuNCs exerted both promotional and inhibitory effects on the fermentation products of *G. lingzhi*.

### Effect of the addition time of gold nanomaterials on the yield of polysaccharides and triterpenoids

The impact of exogenous additives added at different stages during the liquid fermentation of *G. lingzhi* on the biosynthesis of induced secondary metabolites has been reported [52]. In the work of Tang and Zhu et al., the total accumulation of ganoderic acid and exopolysaccharides was increased by adding 1 mM copper ions on days 2, 6, 8, and 2mM copper ions on days 4. Notably, the highest content of ganoderic acid reached a level of 3.0 ± 0.1 mg/100 mg DW. Furthermore, higher concentrations of copper addition on the fourth day resulted in elevated levels of intracellular polysaccharide content and overall yield [53].

Therefore, this study investigates the impact of adding gold nanomaterials at different stages on the production of *G. lingzhi* polysaccharides and triterpenoids, at the most prominent concentration of each gold nanomaterial in comparison to the control group determined in Sect. Effect gold nanomaterials concentration on the yield of polysaccharides and triterpenoids. During the fermentation process, three types of gold nanomaterials were added during lag phase, log phase and stationary phase of mycelial growth. The cultures were then incubated for 8 days before analyzing the fermentation products.

**Fig. 10:**
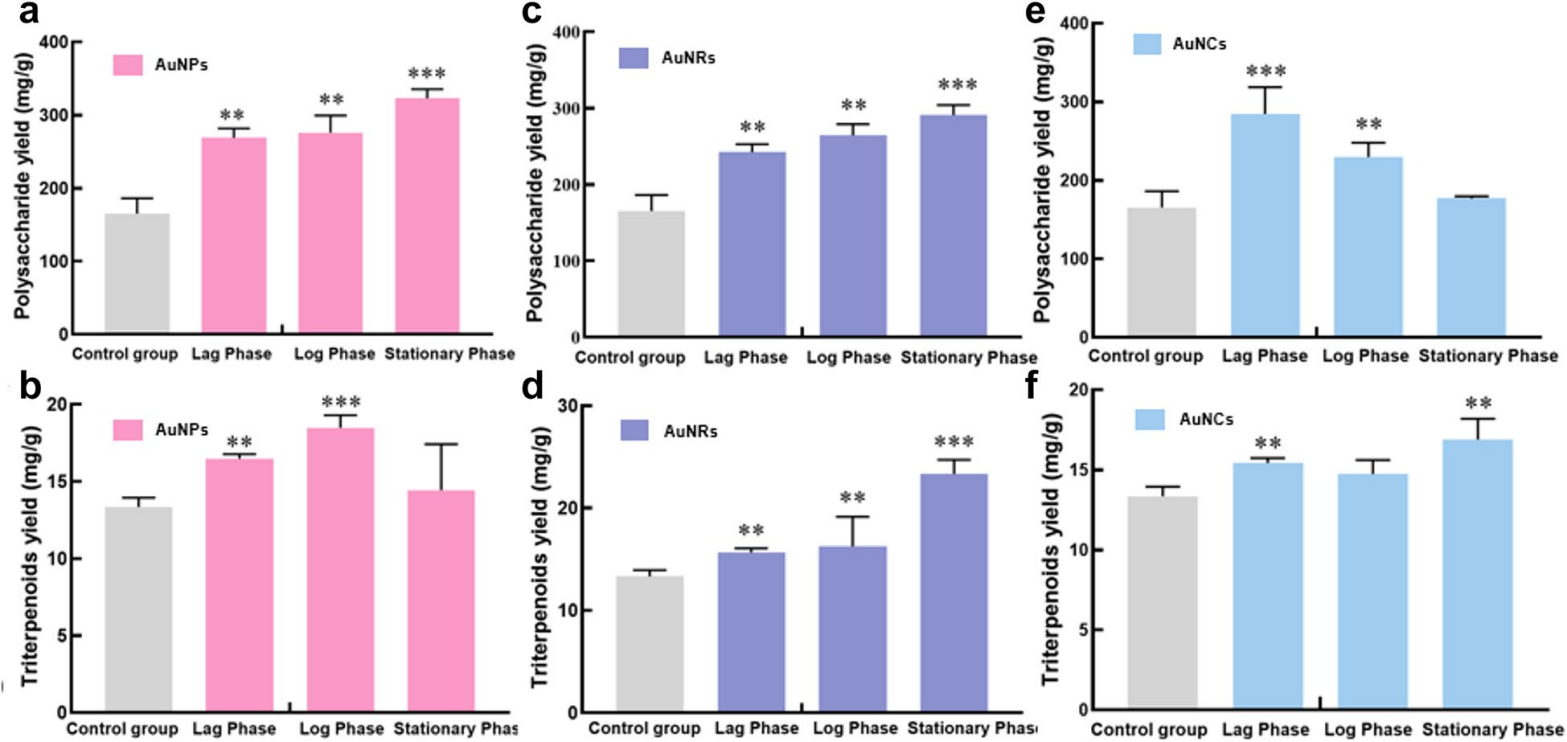
**a** Effect of adding 136 mg/L AuNPs in different periods on the yield of *G. lingzhi* polysaccharide. **b** Effects of adding 6.8 mg/L AuNPs at different stages on yield of *G. lingzhi* triterpenoids. Effect of adding 136 mg/L AuNRs in different periods on *G. lingzhi* fermentation products **c** Polysaccharide yield **d** Triterpenoids yield. **e** Effect of adding 102 mg/L AuNCs in different periods on the yield of G. lingzhi polysaccharide. **f** Effects of adding 68 mg/L AuNCs at different stages on yield of *G. lingzhi* triterpenoids. The data in the figure is the mean ± standard error, * Stands for *P*<0.05, ** stands for *P*<0.01, *** stands for *P*<0.001

As depicted in Fig. 10a, the addition of 136 mg/L AuNPs during the lag phase, log phase, and stationary phase effectively enhances *G. lingzhi* polysaccharide synthesis. Particularly noteworthy is that when added during the stationary phase, the yield of *G. Lingzhi* polysaccharide reached 332.83 mg/g, exhibiting a remarkable increase of 50.37% compared to the control group (165.68 mg/g).

Fig. 10b illustrates the variation trend in *G. lingzhi* triterpenoids content upon the addition of 6.8 mg/L AuNPs at different stages. The results demonstrated that adding AuNPs during the log phase led to a peak yield of *G. lingzhi* triterpenoids at 18.49 mg/g, which represents an improvement of 27.80% over that observed in the control group (13.35 mg/g)

Figure 10c demonstrates that the incorporation of AuNRs at each phase of *G. lingzhi* growth enhances the production of *G. lingzhi* polysaccharide. Remarkably, when 136 mg/L of AuNRs was added during the stationary phase, the highest yield of *G. lingzhi* polysaccharide reached 291.04 mg/g, exhibiting a remarkable increase of 43.08% compared to that observed in the control group. As shown in Fig. 10d, the addition of AuNRs at this stationary phase led to a substantial elevation in triterpenoid content, which reached 23.33 mg/g and exhibited a remarkable enhancement of 42.78% compared to the control.

Based on the analysis of AuNCs addition concentration’s effect on polysaccharide and triterpenoid yield, we determined that the optimal *G. lingzhi* polysaccharide yield was achieved at an AuNCs addition concentration of 102 mg/L, while the highest *G. lingzhi* triterpenoid yield was obtained at 68 mg/L. As shown in Fig. 10e, the addition of AuNCs during both lag and log phase significantly increased the yield of *G. lingzhi* polysaccharide compared to the control group. Particularly, adding AuNCs during the lag phase resulted in a remarkable increase in polysaccharide yield, reaching 284.79 mg/g which was 41.96% higher than that of the control group. Similarly, adding AuNCs during both stationary and lag phases significantly enhanced the production of *G. lingzhi* triterpenoid compared to the control group in the Fig. 10f. Notably, adding AuNCs during the stationary phase led to a triterpene yield of 16.90 mg/g.

By comprehensively considering the morphology, addition concentration, and timing of gold nanomaterials, we observed that adding AuNPs with a concentration of 136 mg/L during the stationary phase resulted in a maximum yield of *G. lingzhi* polysaccharides at 332.83 mg/g, representing a significant increase of 50.37% compared to the control group. Similarly, when AuNRs with a concentration of 136 mg/L were added during the stationary phase, the yield of *G. lingzhi* triterpenoids reached its peak at 23.33 mg/g, showing an improvement of 42.78% compared to the control group.

As one of the pivotal bioactive metabolites in *G. lingzhi*, polysaccharides play a crucial role in cell wall composition. The incorporation of metal nanomaterials in the cell culture process has been demonstrated to enhance immobilized cellulase and cell wall thickening [9, 54], The observed increase in polysaccharide content may result from nanomaterials modulating the expression of biosynthetic genes and regulatory proteins in *G. lingzhi*, including those involved in cell wall regulation, thereby enhancing polysaccharide production.

*G. lingzhi* triterpenes are one of the main chemical and pharmacological components of *G. lingzhi*, and also an important accumulation substance for *G. lingzhi* to develop into a fruiting body. In this study, despite the decrease in *G. lingzhi* biomass, the product yield increased. The addition of nanoparticles may act as an adverse environmental factor during the submerged fermentation of *G. lingzhi*. When exposed to unfavorable conditions, *G. lingzhi* cells initiate stress responses that induce ROS secretion, promoting secondary metabolite accumulation and subsequently affecting triterpenoids production [55, 56]. Based on these mechanisms, we hypothesize that gold nanoparticles in this study may regulate polysaccharides and triterpenoids content through ROS generation.

## Conclusions

In this study, three types of gold nanomaterials with varying sizes, shapes, and forms were synthesized and added to the liquid fermentation of *G. lingzhi* to investigate their effects on its growth. The results indicated that the addition of gold nanomaterials inhibited mycelium growth, resulting in reduced biomass and delayed substrate consumption. However, electronic scanning and fluorescence microscope images revealed negligible cytotoxic effects of gold nanomaterials on the morphology of *G. lingzhi* mycelia. Furthermore, it was found that gold nanomaterials promoted secondary metabolite production during *G. lingzhi* fermentation. Therefore, we further explored the effects of different types, concentrations, and adding time of gold nanomaterials on the fermentation products of *G. lingzhi.* The maximum yield of (333.83 mg/g) for polysaccharides with a 50.37% increase compared to the control group is determined when adding 136 mg/L concentration AuNPs at stationary stage; while adding 136 mg/L concentration AuNRs at stationary phase resulted in maximum yield (23.33 mg/g) for triterpenes with a 42.78% increase compared to control group.

The subsequent step involves further elucidating the ability of smaller AuNCs to traverse the cellular membrane and infiltrate microorganisms, as well as investigating their impact on microbial cell metabolism. Simultaneously, we will conduct comprehensive transcriptome, proteome, and metabolome analyses to investigate the potential impact of these goldnanomaterials on gene expression, protein synthesis, and metabolite accumulation in *G. lingzhi*. Additionally, we will assess the anticancer/anti-inflammatory properties of *G. lingzhi* fermentation products supplemented with gold nanomaterials and observe any resultant effects.

## Supplementary Information

Below is the link to the electronic supplementary material.

Supplementary Material 1

## Data Availability

No datasets were generated or analysed during the current study.
