# Supplementary material for: Effect of different colloidal gold nanomaterials on Ganoderma lingzhi fermentation for production of ganoderma polysaccharide and triterpenoid through macroscopic and microscopic investigation
Source: Microb Cell Fact. 2025 Sep 30;24:211. doi: 10.1186/s12934-025-02810-0 (PMC12487537; doi:10.1186/s12934-025-02810-0)
Supplement: Supplementary file 1 — Supplementary Material 1 [file 12934_2025_2810_MOESM1_ESM.docx]

**Supporting information**

**Effect of Different Colloidal Gold Nanomaterials on *Ganoderma Lingzhi* Fermentation for Production of *Ganoderma* Polysaccharide and Triterpenoid through Macroscopic and Microscopic Investigation**

Mengqiu Luo^a,1^; Muling Shi^a,c,1,^*; Yang Li^a^; Yiqing Yang^b^; Hanqi Wei^a^; Shengwen Luo^a^; Wenhuan Huang^b^; Yida Deng^a^*; Gao-Qiang Liu^b^*

^a^Key Laboratory of Pico Electron Microscopy of Hainan Province; School of Materials Science and Engineering; State Key Laboratory of Tropic Ocean Engineering Materials and Materials Evaluation; Hainan University, Haikou, Hainan Province 570228, China

^b^Hunan Provincial Key Laboratory of Forestry Biotechnology, College of Life Science and Technology, Central South University of Forestry & Technology, Changsha, Hunan Province 410004, China

^c^Molecular Science and Biomedicine Laboratory, State Key Laboratory of Chemo/Biosensing and Chemometrics, College of Chemistry and Chemical Engineering, College of Biology, Hunan University, Changsha 410082, China

*Corresponding authors.

E-mail addresses: [mulingshi@hnu.edu.cn](mailto:mulingshi@hnu.edu.cn) (Muling Shi), [yd_deng@hainanu.edu.cn](mailto:yd_deng@hainanu.edu.cn) (Yida Deng), [gaoliuedu@csuft.edu.cn](mailto:gaoliuedu@csuft.edu.cn) (Gao-Qiang Liu)

1 These authors contributed equally to this work.

1. **Synthesis and characterization of gold nanomaterials**

AuNPs were prepared by sodium citrate reduction method. Add 200 mI of 0.01% HAuCl4 to a 500 ml beaker in a boiling water bath. 3.7 ml 1 % sodium citrate solution was added to boiled 0.01 % HAuCl_4_ rapidly. 3.7 ml of 1% sodium citrate solution was rapidly added to boiling 0.01% HAuCl4, and after 10 minutes of reaction, the solution turned wine red, and after cooling to room temperature, a solution of AuNPs was obtained.

AuNRs were prepared by seed crystal method. 0.12 ml HAuCl_4_ was added to 2.5 ml CTAB solution (0.2 mol / L) and 1 ml ultrapure water, under the condition of continuous stirring, 0.6 ml of freshly configured NaBH_4_ solution was added. After the solution became light brown, it was stirred for 2 min to obtain a seed solution for later use. 88.7 ml CTAB solution (0.1 mol / L) was added to 250 ml conical flask, followed by 4 ml AgNO_3_ solution, 5 ml HAuCl_4_ solution and 1.24 ml ascorbic acid solution. The solution was fully stirred to become transparent and colorless. Then 1 ml of seed solution was added and kept at 27.5 ℃ for 12 h. The solution turned reddish brown, which was the successful preparation of AuNRs solution.

AuNCs were prepared by glutathione reduction. 135.3 ml ultrapure water was added to a 250 ml conical flask, followed by 10.2 ml of 1 % HAuCl_4_, and finally 4.5 ml of 100 mmol / L GSH was added. After mixing for 5 min at room temperature, the mixture was put into a thermostatic water bath at 70 ℃ and reacted for 24 h to obtain the AuNCs solution.

A transmission electron microscope (TEM), Model JEM-2100, Japan, was used to investigate the particle size and morphology of the synthesized samples. scanning electron microscope (SEM) images and were taken on the Tescan Mira4 field emission SEM. Investigation of the structural and composition change of different samples was performed by UV-visible spectrophotometer, Model WFZ-UV-2100. The SEM and TEM sample preparation methods of the three gold nanomaterials are the same.

Scanning electron microscope sample preparation. First, the silicon wafer is repeatedly washed under distilled water, and then placed in ultrasonic cleaning. The instrument was washed for 1 h and stored in anhydrous ethanol. 10 µL of AuNPs solution was dropped onto clean silicon wafers and placed in an oven until the solution was dry.

Transmission electron microscopy. Prepared clean filter paper placed in a glass dish, the copper mesh carefully placed in. The copper mesh is placed in a glass dryer until the copper mesh is completely dried, which is considered that the sample has been prepared.

1. **Determination of amylase activity**

Th The control and the three gold nanomaterials of fermentation were sampled for 8 consecutive days,3 mL of the fermentation broth was taken each time, centrifuged for 10 minutes, and the supernatant was taken as crude enzyme solution. 24 test tubes were taken, 12 for the control group and 12 for the assay group, three technical replicates were set up for each experimental group. Add 1 ml of crude enzyme extract and 1 ml of 0.1 mol/L (pH 5.6) citrate buffer to each tube, and then add 4 ml of 0.4 mol/L NaOH solution to the control group to terminate the reaction. Place each tube in a constant temperature water bath at 40℃ for 15 min, add 2 ml of 2% soluble starch solution preheated at 40℃ to each tube, and immediately put it into the accurate water bath at 40℃ for 5 min, then remove the tubes and quickly add 4 ml of 0.4 mol/L NaOH solution to the assay group to terminate the reaction. Take 2 ml of the enzyme solution from each of the above test tubes, add 2.0 ml of DNS solution, mix thoroughly, boil accurately in a boiling water bath for 5 min, remove the test tubes and rinse under running water to cool the solution, fix the volume to 25 ml with ultrapure water, and mix well. The absorbance was measured at 520 nm, and then the maltose content was calculated by the formula of the maltose standard curve, and the amylase enzyme activity was calculated by the formula, and the final result was based on the average value of three parallel experiments.

$$\text{Amylase activity (μg/min)=C×V×1000×dilution/T}$$

Among them: C is the concentration of maltose (g/L), V is the reaction volume (L), and T is the reaction time (min).

Maltose standard curve determination: Take 21 test tubes of 25 ml graduated test tubes, numbered 1~7, 3 parallel groups each. Add 1 mg/ml maltose standard solution 0, 0.2, 0.6, 1.0, 1.4, 1.8, 2.0 ml, L, respectively, and refill with ultrapure water to 2.0 ml. Add 2.0 ml DNS solution to each tube, boil accurately in a boiling water bath for 5 min, rinse the tubes under running water to cool the solution, and fix the volume with ultrapure water to 25 ml. Using an enzyme marker, determine the absorbance value (A) at 520 nm. The absorbance value (A) was measured at 520 nm using an enzyme marker, the vertical coordinate is the absorbance value, and the horizontal coordinate is the content of maltose to make the standard curve.

1. **Determination of maltose standard curve**

A 25 ml scale test tube was taken and numbered 1-7, with 3 parallels in each group. 1 mg/ml maltose standard solution 0,0.2,0.6,1.0,1.4,1.8,2.0 ml were added respectively, and ultrapure water was added to 2.0 ml. Then 2.0 ml DNS solution was added to each tube, and the boiling water bath was accurately boiled for 5 min. The tube was taken out and rinsed to cool the solution, and the solution was diluted to 25 ml with ultrapure water. The specific operation is shown in table 1. The absorbance value (A) was measured at 520 nm using a microplate reader. The ordinate was the absorbance value, and the abscissa was the maltose content to make a standard curve.

Table 1. Maltose Standard Curve

| reagent | addition | | | | | | |
| --- | --- | --- | --- | --- | --- | --- | --- |
| Maltose | 0 | 0.2 | 0.6 | 1.0 | 1.4 | 1.8 | 2.0 |
| Ultrapure water | 2.0 | 1.8 | 1.4 | 1.0 | 0.6 | 0.2 | 0 |
| DNS | 2.0 | 2.0 | 2.0 | 2.0 | 2.0 | 2.0 | 2.0 |
| bulk volume | 25.0 | 25.0 | 25.0 | 25.0 | 25.0 | 25.0 | 25.0 |

The maltose standard curve is shown in Figure s1, and the linear regression equation is y = 0.6051x - 0.0378. The correlation coefficient R^2^ = 0.991, indicating a good degree of correlation.


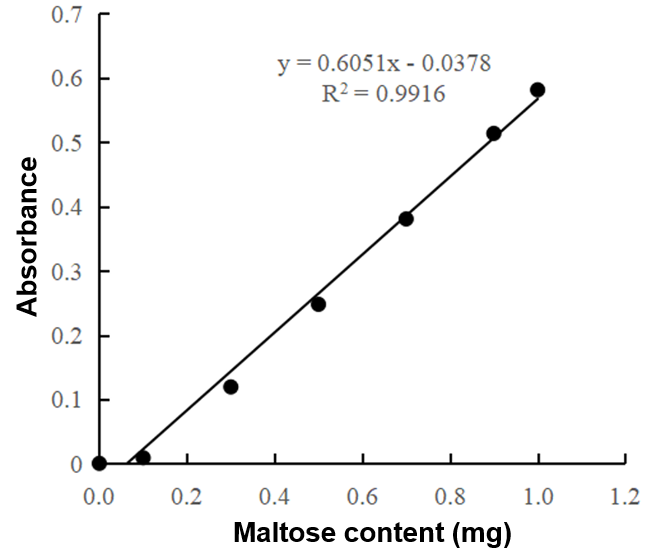


Figure S1. Maltose Standard Curve

1. **Determination of polysaccharide standard curve**

Determination of polysaccharide standard glucose curve; the content of polysaccharides in *G. lingzhi* was determined by the phenol-sulfuric acid method. Take 7 groups of washed test tubes, 3 parallels in each group. Take 0 ml, 0.02 ml, 0.04 ml, 0.06 ml, 0.08 ml, 0.10 ml, and 0.12 ml of the above glucose standard solution in the test tube, and add ultrapure water to 2 ml. Then 1 ml of 6 % phenol solution and 5 ml of concentrated sulfuric acid were added to each test tube. After standing for 10 minutes, they were shaken and waited to cool to room temperature. The absorbance (A) was measured at 490 nm. The specific operation is shown in Table 1.

Table 2. Maltose Standard Curve

| reagent | addition | | | | | | |
| --- | --- | --- | --- | --- | --- | --- | --- |
| Glucose | 0 | 0.02 | 0.04 | 0.06 | 0.08 | 0.10 | 0,12 |
| Ultrapure water | 2.0 | 1.98 | 1.96 | 1.94 | 1.92 | 1.90 | 1.88 |
| 6 % phenol solution | 1.0 | 1.0 | 1.0 | 1.0 | 1.0 | 1.0 | 1.0 |
| H_2_SO_4_ | 5.0 | 5.0 | 5.0 | 5.0 | 5.0 | 5.0 | 05.0 |

The standard curve was drawn with glucose content ( mg ) as abscissa and absorbance as ordinate. As shown in Figure s1, the linear regression equation was y = 4.6965x + 0.0033, and the correlation coefficient R^2^ = 0.999. The results showed that the correlation was good.


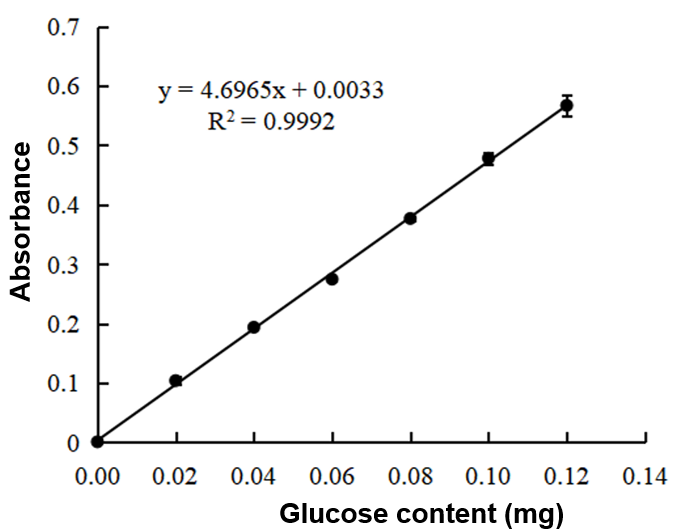


Figure S2. Standard curve of glucose

1. **Determination of triterpenoids standard curve**

Determination of triterpenoids marker (ursolic acid) standard curve; *G. lingzhi* triterpenoids were determined by the vanillin-iceacetic acid method. Eight washed test tubes were taken, each in three parallel. 0 ml, 0.02 ml, 0.04 ml, 0.06 ml, 0.08 ml, 0.10 ml, 0.12 ml, and 0.14 ml of ursolic acid standard solution were taken into each test tube, and the solvent was evaporated under boiling the solvent was evaporated under boiling water. Added 0.4 ml of 5% vanillin-glacial acetic acid solution and 1 ml of perchloric acid to the test tubes, shaken well, and placed in a water bath at 60°C for 15 min. After the water bath was cooled to room temperature, 5 ml of glacial acetic acid was added to each test tube. The absorbance (A) was measured at 548 nm after shaking well and standing for 20 min. The specific operation is shown in Table 2.

Table 3. Standard curve of Ursolic acid

| reagent | addition | | | | | | |  |
| --- | --- | --- | --- | --- | --- | --- | --- | --- |
| Ursolic acid | 0 | 0.02 | 0.04 | 0.06 | 0.08 | 0.10 | 0,12 | 0.14 |
| 5% vanillin-glacial acetic acid | 0.4 | 0.4 | 0.4 | 0.4 | 0.4 | 0.4 | 0.4 | 0.4 |
| CH_3_COOH | 1.0 | 1.0 | 1.0 | 1.0 | 1.0 | 1.0 | 1.0 |  |
| H_2_SO_4_ | 5.0 | 5.0 | 5.0 | 5.0 | 5.0 | 5.0 | 05.0 |  |

The standard curve was drawn with ursolic acid content (mg) as abscissa and absorbance as ordinate, as shown in Figure s2. The linear regression equation is y = 2.3556x + 0.0128, the correlation coefficient R2 = 0.990, the results show that the correlation.


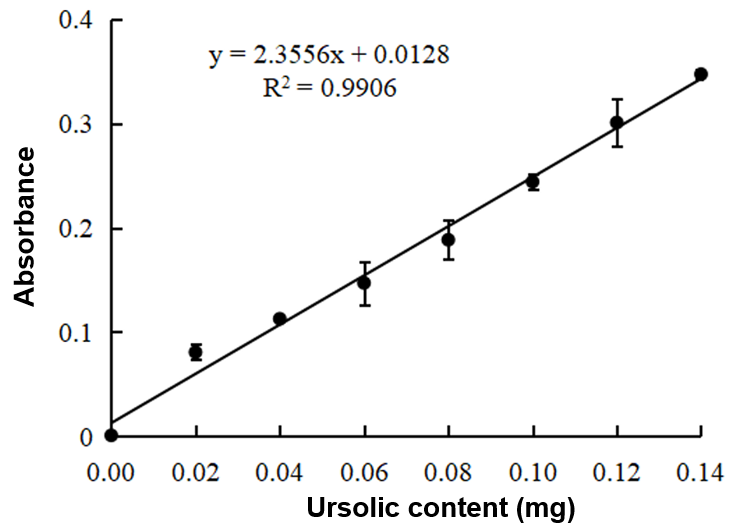


Figure S3. Standard curve of Ursolic acid


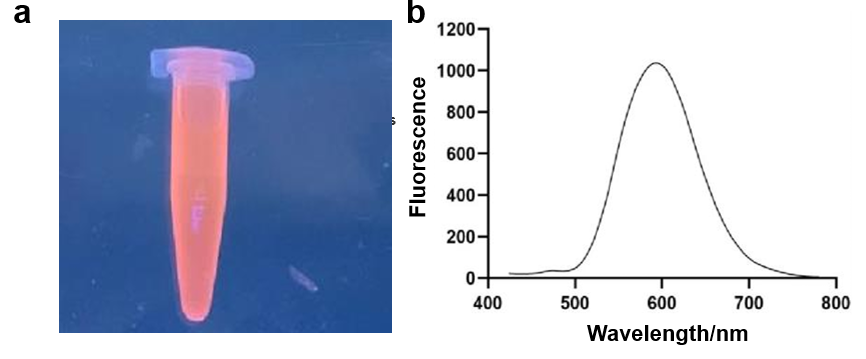


Figure S4. (a) Gold nanoclusters under Ultraviolet Light (b) Fluorescence spectra of gold nanoclusters


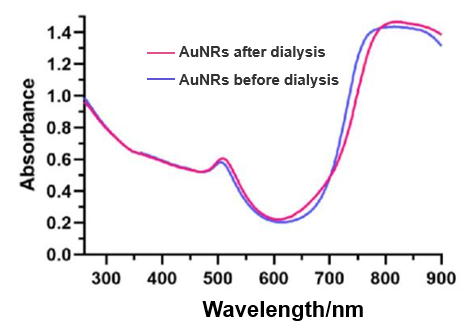


Figure S5. UV absorption spectra of gold nanorods before and after centrifugation





Figure S6. Changes of the percentage of DO in G. lingzhi fermentation broth. The addition concentrations for all three types of gold nanomaterials were all set at 136 mg/L. All data points represent the mean ± SD of three independent technical replicates.


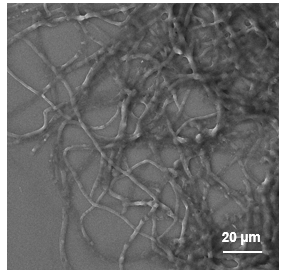


Figure S7. SEM image of *G. lingzhi* mycelium at 2000× magnification

Table 4. Element content of *G. lingzhi* spectrum18 site (Wt%: Mass percentage; Atomic%: Element content or atomic content.）

| Element | Wt% | Wt% Sigma | Atomic% |
| --- | --- | --- | --- |
| C | 49.10 | 0.53 | 67.49 |
| O | 5.80 | 0.23 | 5.98 |
| Na | 0.10 | 0.03 | 0.07 |
| Si | 45.01 | 0.47 | 26.46 |
| Total | 100.00 |  | 100.00 |

Table 5. Element content of spectrum9 site

| Element | Wt% | Wt% Sigma | Atomic% |
| --- | --- | --- | --- |
| C | 49.27 | 0.52 | 69.45 |
| O | 4.73 | 0.22 | 5.01 |
| Na | 0.39 | 0.04 | 0.29 |
| Si | 41.29 | 0.43 | 24.89 |
| Au | 4.32 | 0.23 | 0.37 |
| Total | 100.00 |  | 100.00 |

Table 6. Element content of *G. lingzhi* spectrum15 site

| Element | Wt% | Wt%Sigma | Atomic% |
| --- | --- | --- | --- |
| C | 43.24 | 0.63 | 64.08 |
| O | 3.33 | 0.21 | 3.71 |
| Si | 50.40 | 0.57 | 31.94 |
| Au | 3.02 | 0.24 | 0.27 |
| Total | 100.00 |  | 100.00 |

Table 7. Element content of *G. lingzhi* spectrum7 site

| Element | Wt% | Wt%Sigma | Atomic% |
| --- | --- | --- | --- |
| C | 49.27 | 3.30 | 64.29 |
| O | 39.44 | 2.99 | 33.81 |
| Na | 3.05 | 1.73 | 1.82 |
| Au | 1.21 | 3.34 | 0.08 |
| Total | 100.00 |  | 100.00 |
